# Studies on Flowability, Compressibility and *In-vitro* Release of*Terminalia chebula* Fruit Powder Tablets

**Published:** 2011

**Authors:** Singh Satya Prakash, Niranjan Patra, Chakraborty Santanu, Pandit Hemant Kumar, Jagannath Patro, Vimala Devi

**Affiliations:** *Division of Formulation and Development, PG Department of Pharmaceutics, College of Pharmaceutical Sciences, Berhampur, Orissa, India. *

**Keywords:** *Terminalia chebula*, Flowability, Compressibility, Dissolution.

## Abstract

The dried fruit of *Terminalia chebula *is widely used for its laxative properties. The objective of the present study was to examine the flowability and compressibility of *Terminalia chebula *fruit powder, subsequently developing its tablet formulations by utilizing wet granulation and direct compression technology. Initial studies on flowability and compressibility revealed that the fruit powder flows poorly, is poorly compressible and mucilaginous in nature. The consolidation behaviors of the fruit powder and of its tablet formulations were studied using the Kawakita, Heckel and Leuenberger equations. Kawakita analysis revealed reduced cohesiveness hence improved flowability was achieved in formulations prepared by direct compression and the wet granulation technique. The Heckel plot showed that the *Terminalia chebula *fruit powder when formulated using direct compression showed initial fragmentation followed by plastic deformation and that the granules exhibited plastic deformation without initial fragmentation. The compression susceptibility parameter obtained from the Leuenberger equation for compacts formed by using the direct compression and wet granulation techniques indicated that the maximum crushing strength is reached faster and at lower compression pressures. The Tannin content (with reference to standard tannin) in fruit powder and tablet formulations was determined by UV spectrophotometry at 273 nm. The *in-vitro *dissolution study in simulated SGF (without enzymes) showed more than a 90% release of tannin from the tablets with in 1 h. The brittle fracture index value revealed that tablets prepared from granules showed less fracture tendency in comparison to those formed by direct compression formulation. From this study, it was concluded that the desired flowability, compressibility and compactibility of *Terminalia chebula *fruit powder can be obtained by using the direct compression and wet granulation techniques.

## Introduction

*Terminalia chebula *fruit is an ingredient of triphala, a well known formulation of ayurveda used as a laxative, hypolipidaemic and antioxidant agent (1, 2). *Terminalia chebula *fruit has also been reported to have adaptogenic (3) and cardiac activities (4), in addition to antiviral and antibacterial (5) actions. *Terminalia chebula *fruits are an important source of tannin. The tannins of Haritaki are of the pyrogallol type (hydroysable tannins), which on hydrolysis yield chebulic acid and d-galloy glucose. Chebulagic, chebulinic, ellagic, and gallic acid are other components of the fruit. It also contains glucose and sorbitol (about 3.5 %). Resin and a purgative principle of the nature of anthraquinone, and sennosides are present in the fruit. 

In spite of their efficacy, herbal medicinal products have been widely criticized due to a lack of standardization and poor quality presentation. Formulation of *Terminalia chebula *fruit powder into a measured tablet form could ensure dosage precision. Moreover the formulation of *Terminalia chebula *fruit powder into the form of a modern pharmaceutical tablet would mean it benefiting from the useful properties tablets have. The benefits of tabletizing *Terminalia chebula *fruit powder include ease of administration, greater acceptance due to presentation, prolonged shelf life, quality assurance, greater accuracy in dispensing and reduction in transportation costs (6). Therefore, with a basic understanding of the initial physical and micromeritic properties of *Terminalia chebula *fruit powder more data could be generated to optimize the tabletizing properties of the fruit powder. Hence, the objective of the present study is to produce conventional tablets from *Terminalia chebula *fruit powder for oral administration using wet granulation and direct compression methods. Subsequently preparing the tablets following a systematic study on flowability and compressibility (7) with the aim of characterizing consolidation behavior.

## Experimental

*Materials*

The standard tannin sample used in the experiment was obtained as gratis from Sami Labs, India. Avicel PH 101, Avicel PH 102 and the cross povidone specimen was obtained as gratis from Ranbaxy Laboratory Ltd, India. All other chemicals used were of analytical grade.

*Methods*

*Collection of plant material*

The fruits of the *Terminalia chebula *plant were collected from the campus of the College of Pharmaceutical Sciences, Mohuda, India and were identified, then authenticated by Botanist, PG Dept of Biosciences, Berhampur University, India. The voucher specimen (0158/07/PGDB/BU) was deposited in the University’s repository herbarium for future reference. The collected fruits were shade dried, powdered and passed through a no. 85 sieve.

*Determination of quantitative standards and drug content*

The *Terminalia chebula *fruit powder was subjected to various quantitative tests such as acid insoluble ash, total ash, foreign organic matter, alcohol soluble extractive and water soluble extractive, and then compared with official standards (8). The average tannin content was determined by using a UV-Visible spectrophotometer (UV-2450, Shimadzu, Japan) at 273 λ_max_ nm (9).

*Preparation of granules*

The wet granulation method of massing and screening was utilized for a batch size of 1000 tablets. *Terminalia chebula *fruit powder (86% w/w), Avicel pH101 (10% w/w) and cross povidone (3% w/w) were dry mixed in a Wet Granulator WGS (Kalweka Series, Karnavati Engineering Ltd, India). The dry mix was moistened with an appropriate amount of granulating liquid that being 90% alcohol (v/v) and subjected to wet mixing in the identical wet granulator. The wet mass was passed through a No. 16 sieve. The resulting granules were dried in a Hot Air Oven (Hicon India Ltd, India) for 4 hrs at 60°C and then re-sieved through a No.16 sieve. Talc and magnesium stearate (1 % w/w) were added to the granules and then subsequently mixed for 4 min in a Cube mixer (Kalweka series, Karnavati Engineering Ltd, India).

*Preparation of a direct compression formulation*

In the direct compression method, *Terminalia chebula *fruit powder (77% w/w), Avicel pH102 (18% w/w), cross povidone (3% w/w) and talc (2% w/w) were mixed in a Cube mixer (Kalweka series, Karnavati Engineering Ltd, India) for a batch size of 1000 tablets.

*Fundamental powder and granule properties Bulk and tap density*

The bulk and tap density of *Terminalia chebula *fruit powder and its formulations was determined by the tapping method (n = 10) using digital tap density apparatus (Electro lab ltd. India).

*Flow rate*

The flow rate (10) (Karsten and Katharina, 2004) of the *Terminalia chebula *fruit powder and its formulations were determined as the ratio of mass (g) to time (sec) using a steel funnel with an orifice diameter of 10 mm (n = 10).

*Kawakita analysis*

Flowability was determined using the Kawakita analysis (11). The method involved pouring 10 g of powder and its formulations into a 50 mL glass measuring cylinder. The heaped particles in the cylinder were then leveled off horizontally with a thin metallic spatula and the bulk volume *Vo *was accurately measured. Tapping was afterwards initiated mechanically and the change in volume of the powder column *V*_N_ was noted after *N *no of taps. The behavior of both the powder and its formulations in the tapping procedure were compared using numerical constants obtained from Kawakita plots.

The Kawakita equation, which is used for assessing the flow properties of powders, is given by:

$\frac{N}{C}=\frac{N}{a}+\frac{1}{\mathrm{ab}}$                      (2)

Where *a *and *b *are constants; *a *describes the degree of volume reduction at the limit of tapping and is called compactibility; *1/b *is considered to be a constant related to cohesion and is called cohesiveness, *C *being the degree of volume reduction is calculated from the initial volume *V*_0_ and tapped volume *V*_N_ as:

$C=\frac{\left(V_{0}-V_{N}\right)}{V}$                     (3)

The numerical values for constants *a *and *1/b *are obtained from the slope of the plot of *N/C *versus number of taps *N (N = *10, 20, 30 up to 300*)*.

*Compaction studies*

*Preparation of compacts*

Compacts containing 500 mg of *Terminalia chebula *were made using the fruit powder and its formulations, using a Hydraulic pellet press (Kimaya Engineers, India). Compression loads were used; ranging from 10Kg/cm^2^ to 95Kg/cm^2^. Ten compacts were made at each compression level. Before compression, the die (13mm diameter) and the flat-faced punches were lubricated using a 2% w/v dispersion of magnesium stearate in ethanol ether (1:1). The compacts were stored over silica gel for 24 h (to allow for elastic recovery and hardening and prevent false low yield values) before evaluations. The dimensions (thickness and diameter) and weight uniformity of three compacts were determined. The relative density *ρ*_r _was calculated as the ratio of apparent density *ρ*_A_ of the compact to the true density *ρ*_T_, of the powder. The data obtained using this ‘ejected tablet method’ was used to obtain Heckel plots. Linear regression analysis was carried out over a compression range of between 10Kg/cm^2^ and 95Kg/cm^2^ then the parameters using Heckel plots (12) were calculated.

*Heckel equation*

The compaction characteristics of the powder were studied by means of the Heckel equation.

$\mathrm{In}\frac{1}{1-P_{r}}=\mathrm{KP}+A$                     (4)

$\rho_{r}=\frac{\rho_{A}}{\rho_{T}}$                     (5)

Where, *ρ*_r_ is the relative density of the compact, *ρ*_T_ is the apparent density and *ρ*_T_ is the true density, *P *is the applied pressure; *K *(the slope of the linear portion) is the reciprocal of the yield pressure, *Py*, of the material. The yield pressure is inversely related to the ability of the material to deform plastically under pressure and *A *is a function of the original compact volume.

*Leuenberger equation*

For compactibility assessment, the force required for diametral breaking of the compacts was determined using a Digital hardness tester EH-01 (Electro lab ltd. India). The tensile strength σ_χ _of the compacts was calculated using the following equation (13) where, χ is hardness (in Kg/cm^2^), *d *and *t *are the diameter and thickness of the compacts (in mm), respectively.

$\sigma_{x}=\frac{2x}{\mathrm{\pi dt}}$                    (6)

Leuenberger analysis was performed by fitting the data in the following equation (14). A nonlinear plot of tensile strength with respect to product compaction pressure *P *and relative density *ρ*_r_ was obtained using statistical software (Graph Pad Prism4). Where, σ_x __max _is the maximum tensile strength (kg/cm^2^) when *P *will be infinite and *ρ*_r _will be equal to 1, and *γ *is the compression susceptibility.

 σ_x_ *=* σ_x__max_ (1 - *e *^-ρ^*r* ^× ^^γ ^^×^^P^*)*                     (7) 

*Preparation of tablet*

Tablets containing 500 mg of *Terminalia chebula *fruit powder were produced by compressing granules using a single station tablet punching machine (Cadmach Machinery Co Pvt. Ltd., India) equipped with 13 mm circular, flat and plain punches.

*Determination of brittle fracture index*

The crack theory can be used to develop a quantitative expression to measure the brittle fracture tendency (15). The BFI values of the resulting tablets were obtained from the expression (16).

$\mathrm{BFI}=0.5(\frac{T}{T_{0}}-1)$                     (8)

Where To and T are the tensile strengths of tablets with and without a central hole, respectively. The centre hole (≤ 01.2 mm) is a built-in model defect to simulate the actual void formed in the tablet during compression. For a brittle fracture to occur, the ratio T/To = 3. By subtracting 1 and multiplying by 0.5 the maximal BFI value is 1 (unity). The BFI value thus has a range of 0 (no fracture tendency) to 1 (maximal fracture tendency). Tablet samples with BFI values (≥ 0.5) display a high fracture incidence during actual tabletizing. 

*Quality control tests for tablets*

The prepared tablets made using *Terminalia chebula *fruit powder via wet granulation and direct compression were subjected to standard tablet quality control tests (17). Weight variation was determined by weighing 20 tablets individually, the average weight was calculated and the percentage variation of each tablet was determined. Hardness was determined by testing 6 tablets from each formulation using a Digital tablet hardness tester (Electrolab Pvt. Ltd., India) and the average applied pressure (Kg/cm^2^) required to crush each tablet was determined. Friability was determined by firstly weighing 10 tablets then placing them in a friability tester (Electrolab Pvt. Ltd., India), which was rotated for 4 min at 25 rpm. After dusting, the total remaining weight of the tablets was recorded and the percentage of friability was calculated. The disintegration time for the tablets was determined in 900 mL of distilled water using Disintegration test apparatus (Electrolab Pvt. Ltd., India).

**Figure 1:**
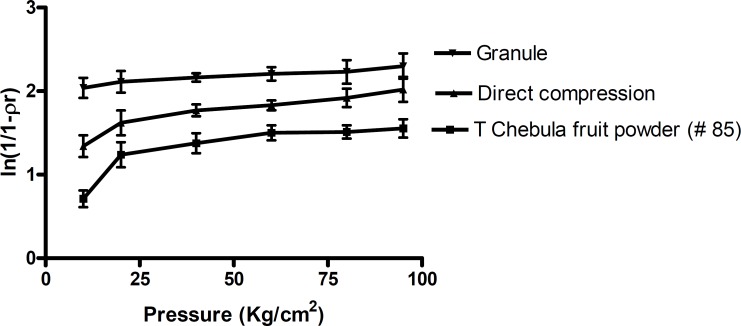
Heckel plot for *Terminalia chebula fruit *powder and its formulations

**Figure 2a:**
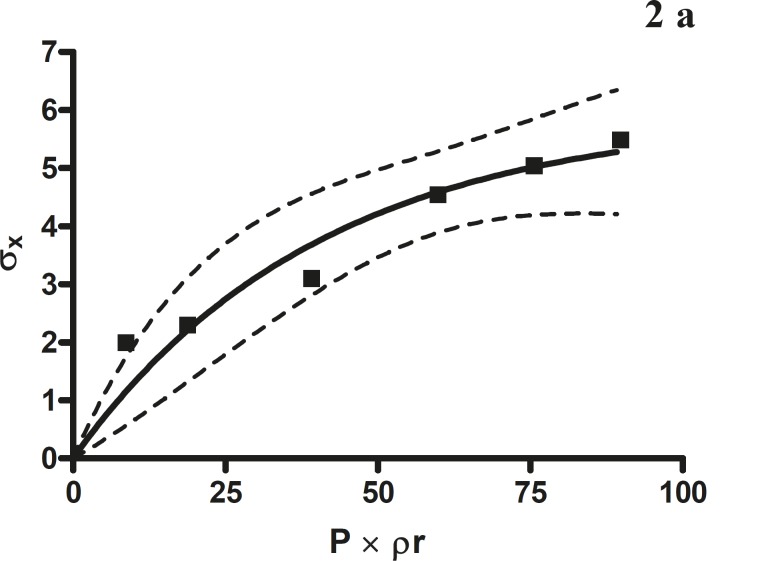
The radial crushing strength was plotted against the product of the pressure of compression and the relative density of *Terminalia chebula *fruit powder

**Figure 2b:**
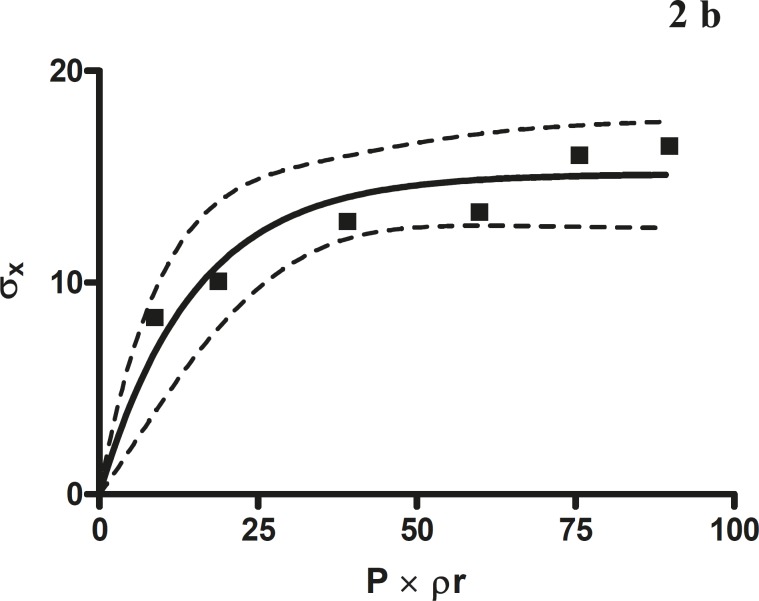
The radial crushing strength was plotted against the product of the pressure of compression and the relative density of *Terminalia chebula *fruit direct compression formulation

**Figure 2c:**
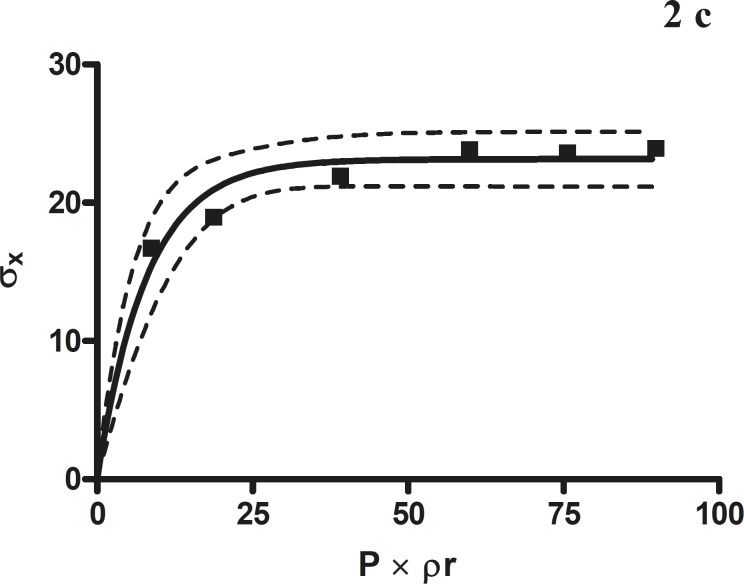
The radial crushing strength was plotted against the product of the pressure of compression and the relative density of the *Terminalia chebula *fruit granules

**Figure 3:**
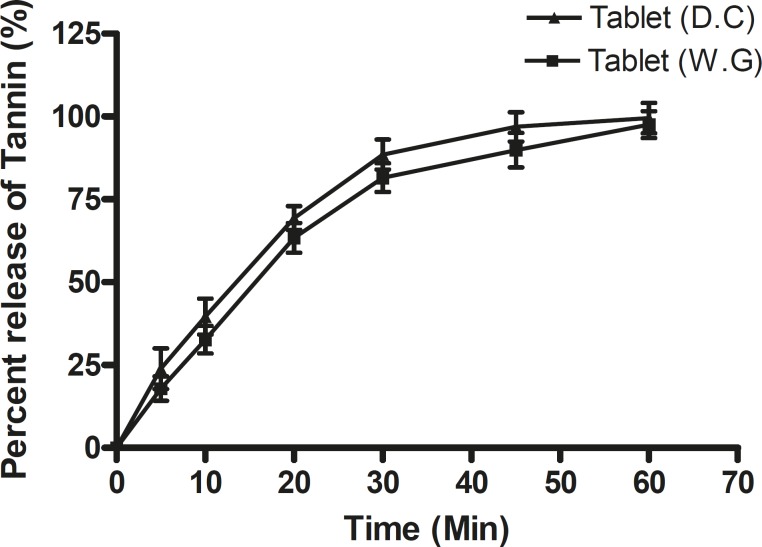
Dissolution profile of tablets prepared by the wet granulation method (Tablet WG) and direct compression method (Tablet DC) in simulated gastric fluid (SGF).

*In-vitro dissolution test*

The release of pure tannin from conventional tablets (18) made from *Terminalia chebula *fruit powder was determined using USP (XXI) six stage dissolution rate test apparatus I (Thermolab^®^) at 50 rpm. Dissolution was examined using 900 mL of 0.1M HCl. The temperature was maintained at 37 ± 0.2°C. Samples each containing 5 mL were withdrawn at 5, 10, 20, 30, 40, 50 and 60 min time intervals, filtered through a Whatman filter (0.45 μm) (Auroco Pvt Ltd, Thailand) and replaced with an equal amount of fresh dissolution medium. Samples were then suitably diluted and analyzed for tannin content using a UV/Visible double beam spectrophotometer (UV-2450 Shimadzu Japan) at 273 nm respectively. The amount of tannin was calculated from the calibration curve of standard tannin. The release studies were conducted in triplicate**. **

*Statistical analysis*

Statistical analysis was carried out to find the differences that exist among the fruit powder and its formulations prepared by wet granulation and direct compression for each of the parameters as shown in Table 1-4. This was achieved by carrying out a one-way ANOVA at p < 0.05 level using software GraphPad Prism ® 4 (GraphPad Software Inc. San Diego, USA). At a 95% confidence interval, a calculated f-value of more than the critical f-value was considered as being significant. A paired t test was carried out to find out significant difference between release pattern among various formulations *i.e. *wet granulation and direct compression At a 95% confidence interval, t*-*values less than or equal to critical t-value were considered significant.

**Table 1:** Fundamental powder characteristics

**Materials **	**Bulk density (g/cm**3**)**	**Tap density (g/cm**3**)**	**Flow rate (g/sec) **
Powder (# 85)	0.45 ± 0.065	0.55 ± 0.036	No flow
Direct compression	0.435 ± 0.15	0.476 ± 0.049	2.36 ± 0.943
Granule	0.382 ± 0.017	0.439 ± 0.25	4.56 ± 0.572
f-value (F critical )	5820.66* 5.1432	2328.77* 5.1432	4736.71* 7.7086

All values are expressed as mean ± SD, n = 10, * ANOVA at p < 0.05 level showed a significant difference

**Table 2:** Parameters of kawakita analysis

**Kawakita**	**Compactibility (a)**	**Cohesiveness (1/b)**	**Coefficient of determination (r** ^2^ **)**
Powder (# 85)	0.2374 ± 0.025	12.082 ± 1.34	0.996
Direct compression	0.1856 ± 0.012	1.435 ± 0.32	0.998
Granule f-value (F Critical)	0.1313 ± 0.014 2496.33* 5.1432	2.835 ± 0.27 36573.56* 5.1432	0.995

All values are expressed as mean ± SD, n = 10, * ANOVA at p < 0.05 level showed a significant difference

**Table 3:** Parameters of heckel analysis

**Heckel**	**Slope (K)**	**Intercept (A)**	**Yield pressure (P**_y_ **)**	**Coefficient of determination (r** ^2^ **)**
Powder (# 85)	0.042 ± 0.023	0.049 ± 0.045	20.161 ± 1.223	0.854
Direct compression	0.112 ± 0.034	1.243 ± 0.047	9.074 ± 0.43	0.940
Granule	0.181 ± 0.021	2.866 ± 0.12	5.512 ± 0.23	0.911
f-value	43474.33*	17992.88*	15439.29*	
(F Critical)	5.1432	5.1432	5.1432	

All values are expressed as mean ± SD, n = 10, * ANOVA at p < 0.05 level showed a significant difference

**Table 4:** Parameters of leuenberger analysis

Leuenberger	Compression susceptibility γ (1/kg/cm^2^)	Maximum tensile strength σ_x__max _(kg/cm^2^)	Coefficient of determination (r^2^)
Powder (# 85)	0.03138 ± 0.002	5.486 ± 1.36	0.9641
Direct compression	0. 089 ± 0.011	16. 45 ± 0.342	0.933
Granule	0.172 ± 0.004	23.91 ± 2.43	0.9115
f-value	67600.2	11539.45	
(F Critical)	5.1432	5.1432	

All values are expressed as mean ± SD, n = 10, * ANOVA at p < 0.05 level showed a significant difference

**Table 5:** Release profile of tablets prepared by direct compression and wet granulation

**Time (min)**	**Tablet (W.G)**	**Tablet (D.C)**	t-value		t critical
0	0.00 ± 0.00	0.00 ± 0.00			
5	17.91060 ± 3.7	23.91060 ± 6.1			
10	32.66076 ± 4.2	39.66076 ± 5.4			
20	63.35762 ± 4.5	69.35762 ± 3.6	0.0034		2.4469
30	81.53493 ± 4.3	88.53493 ± 4.5			
45	89.87599 ± 5.2	96.87599 ± 4.4			
60	97.56000 ± 4.1	99.56000 ± 4.6			

All values are expressed as mean ± SD, n = 6, * ANOVA at p < 0.05 level showed a significant difference

## Results and Discussion

*Quantitative standards and drug content*

The fruit powder confirmed to the quantitative specifications of *Terminalia chebula *as per Indian herbal Pharmacopoeia. The parameters acid insoluble ash (1.3 ± 0.25%), total ash (3.6 ± 1%), alcohol soluble extractive (45 ± 1.5%) and water soluble extractive (92.65 ± 2.5%) were within the official limits (8). The average tannin content in 100 mg of fruit powder was 74 mg ± 7% calculated as tannin with reference to the tannin reference standard at 273 nm using UV-Vis spectrophotometer (UV-2450, Shimadzu, Japan) (9). 

*Fundamental powder and granule properties*

The fundamental flow properties of the *Terminalia chebula *fruit powder exhibited no flow when passed through a funnel, which revealed that it was not up to the theoretical level for processing into tablet dosage form. As the fruit powder was mucilaginous in nature granulating fluid (Alcohol 80% v/v) was found to be suitable for obtaining optimized altered particle size. The flow rate of direct compression formulation and granules revealed a significant improvement in flowability (Table 1). One of the most important factors affecting the bulk density of a powder and its flow properties is the interparticulate interaction (10). Desirable micromeritic properties and the optimal presence of water diminish the cohesiveness of the powder, resulting in an increased bulk density for granule and direct compression formulations revealing enhanced flowability (19). Similarly, increased tapped density for granule and direct compression formulations indicated a better degree of compactibility as a function of applied pressure (20) (Table 1). 

*Kawakita analysis*

Plots of *N/C *versus *N *(Kawakita plots) for *Terminalia chebula *fruit powder, direct compression formulation and granule, resulted in a linear relationship. Kawakita constants indicate the behavior of the powder from the bulk density state to the tap density state. The constants of the Kawakita equation were resolved from the slope and the intercept of the line from graphs *N/C *versus *N *(Table 2). Granules densified the least (small compressible value) but attained a final packing state slowest of all. The lower value of *a *for the granules revealed better flowability than direct compression formulation. Whereas, a lower value of *1/b *for direct compression formulation showed that it is less cohesive than granules (21).

*Compaction properties*

*Heckel analysis*

The Heckel plots (Figure 2) for the direct compression formulation and granules showed no linearity at early stages of compression, because of particle rearrangement and initial fragmentation. Granules showed highest die filling value in initial stages of rearrangement as indicated by their intercept *A *values. These features of the latter could result in the formation of bridges and arches, which could in turn prevent the close packing of particles in the bulk state. A higher value of *A *for the granules implies a higher degree of fragmentation. At low pressure, the large granules were fractured into small ones, which facilitated further rearrangement. When the compression pressure increased, granules showed plastic deformation (22). Greater slopes indicate a greater degree of plasticity hence better compressibility of the material.

*Terminalia chebula *fruit powder was more resistant to movement, once the initial phase of packing (as a result of die filling) was completed. This could be attributed to the high cohesive forces likely to be present as a result of its amorphous nature. The mean yield pressure, *Py*, values were found to be lower for the granules (Table 3). The results therefore indicated that the granules underwent plastic deformation more easily than the direct compression formulation. This also confirmed that direct compression formulation is somewhat resistant to deformation. 

*Leuenberger equation*

The compression susceptibility parameter (Figure 2c) for the compact formed by the wet granulation technique indicated that the maximum crushing strength is reached faster at lower pressures of compression as opposed to that of the *Terminalia chebula *fruit powder (Figure 2a). A higher value for σ_x__max_ was observed in the case of granules than in the direct compression formulation (Table 4). It showed that granules can build a compact with a higher strength than the direct compression formulation (Figure 2b). A lower value of compression susceptibility for *Terminalia chebula *fruit powder demonstrated that a maximum tensile strength could be obtained slowly at higher pressure.

The parameter σ_x__max_ and compression susceptibility allow a characterization of the different materials (23). A low σ_x__max_ value for *Terminalia chebula *fruit powder indicates poor bonding properties. In this regard *Terminalia chebula *formulations showed moderate bonding properties (Table 4). *Terminalia chebula *fruit powder showed an increasing deviation of different values with regards to radial crushing strength when a higher pressure of compression was applied. Whereas the crushing strength appears to remain constant independent of the increasing pressure of compression for both direct compression and wet granulation. This circumstance can be used as an indication of capping tendency as with increasing compression pressure different internal tensions are generated, which can manifest differently when the crushing strength is determined. This tendency can be confirmed from the fact that it was not possible to produce intact tablets at higher compression pressures because of immediate capping in the die.

*Tablet quality control tests*

All of the tablet batches were produced under similar conditions to avoid processing variables. The weight variation of the *Terminalia chebula *tablets prepared by the wet granulation and direct compression methods were in the range of 550 ± 14 mg and 650 ± 16 mg respectively. The hardness of the tablets was higher for tablets prepared via the wet granulation method (5.2 ± 1.23 kg/cm^2^) than the direct compression method (4.8 ± 2.31 Kg/cm^2^). The thickness of the tablets prepared by wet granulation and the direct compression method was 3.4 ± 0.06 and 3.9 ± 0.04 mm respectively. The percentage friability for tablets prepared by the direct compression method (0.75 ± 0.28%) was more than for tablets prepared by the wet granulation method (0.46 ± 0.16%). The values of the hardness test and percentage friability indicate that the prepared *Terminalia chebula *tablets have good handling properties. Disintegration time was 10 ± 2.5 min and 12 ± 1.5 for tablets prepared by the direct compression and wet granulation methods respectively. Initially when the tablet was prepared with starch as a disintegrating agent, the tablet did not disintegrate within 15 min rather the tablet remained as a compact mass for a period of 40 min. The same problem was observed when pre-gelatinised starch and ac-di- sol were used as disintegrants. However, tablets prepared with cross povidone disintegrated within 15 min due to the capillary action of cross povidone (24).

*In-vitro dissolution study*

More than 90% of the tannin content of tablets prepared by the wet granulation and direct compression techniques was released within 1 hour (Figure 3). 

*Statistical analysis*

Statistical analysis using a one-way ANOVA test showed that the calculated f-value was much higher than the critical f-value for all the parameters at a p < 0.05 significance level. Hence there was a significant improvement in the flowability and compressibility. The t-test showed that the calculated t-value was less than the critical t-value for the release pattern among the different formulations showing a significant difference at p < 0.05 significance level. 

*Brittle fracture index*

The B.F.I. value for the tablets was 0.372 and 0.498 for the wet granulation and direct compression methods respectively. The B.F.I value revealed that the tablets have lesser fracture, capping and lamination tendencies (25). 

In conclusion, both wet granulation and direct compression methods could be used successfully to develop tablet formulations of *Terminalia chebula *fruit powder. Hence, the present study recommends the need to generate similar data for different herbal drugs or ayurvedic formulations, as it is greatly essential in industrial applications. 
